# Effects of Proton Pump Inhibitors on Lung Cancer Precise Radiotherapy-Induced Radiation Pneumonitis

**DOI:** 10.1007/s12013-014-0030-5

**Published:** 2014-05-24

**Authors:** QiaoLi Su, Duoning Wang, Bo Yuan, Feng Liu, Yi Lei, Shuangqing Li

**Affiliations:** Department of General Medicine, West China Hospital, Sichuan University, Chengdu, 610041 People’s Republic of China

**Keywords:** Proton pump inhibitor, Lung cancer, Radiation pneumonitis

## Abstract

The objective of this study was to explore the effects of proton pump inhibitors (PPIs) on the development and prognosis of lung cancer precise radiotherapy-induced radiation pneumonitis. Clinical materials of 84 lung cancer patients who had radiation pneumonitis after precise radiotherapy were retrospectively analyzed, and the patients were divided into PPI group and control group, according to whether or not PPIs were applied. The development and prognosis of patients and the effects of different doses of PPI on patient condition from two groups were compared. There were 57 PPI cases in PPI group and 27 cases in control group. Basic characteristics of patients were not statistically different between the two groups; however, white blood cell count, oxygenation indexes, blood gas pH, and lung imaging index were significantly different (*p* < 0.05), indicating that radiation pneumonitis tended to be more severe in PPI group. As regards effects of PPI on prognosis of two groups, remission rate of radiation pneumonia in PPI group was significantly less than that of the control group. Among 57 cases in PPI group, there were 31 patients applied with PPI ≤ 1DDD and 31 patients applied with PPI > 1DDD. In comparison of the various parameters of patients, 7 days after being applied with different doses of PPI, there were no significant differences between the parameters of radiation pneumonitis. PPIs should be cautiously utilized to avoid the effects of lung cancer radiotherapy-induced radiation pneumonia.

## Introduction

Lung cancer is the leading cause of cancer-related mortality, with 1.6 million (13 %) newly diagnosed patients of total cases and 1.4 million (18 %) deaths worldwide in 2008 [1]. Current treatment options include surgical resection, chemotherapy, and radiation. Although these treatment modalities are proven to be effective in improving the survival rate, they are all associated with various adverse effects which could reduce the quality of life and lead to unsatisfactory prognosis.

Radiation pneumonitis (RP) is the one of the most significant complications of acute treatment-related toxicities in lung cancer. It occurs in 5–15 % of people who go through radiation therapy for lung cancer. The occurrence is higher if chemotherapy is given at the same time [2, 3].

The adverse effect of anti-cancer treatments on digestive system is commonly seen in lung cancer patients. Both radiation and chemotherapy can induce gastrointestinal injury. Incidence of radiation esophagitis is associated with higher radiation dose and concurrent chemotherapy [4]. Chemotherapy has been known to cause various effects on digestive system including nausea, vomiting, loss of appetite, indigestion, etc. Therefore, proton pump inhibitors (PPIs) are frequently used in clinics to provide symptomatic relief.

Recently, researchers have shown that the application of PPIs is a risk factor for community-acquired pneumonia and hospital-acquired pneumonia [5, 6]; however, there is no related evidence regarding the effect of PPIs on RP. In our study, we retrospectively analyzed 84 lung cancer patients who developed RP after precise radiotherapy over a five-year period (2008–2013) to explore the effect of PPIs on the development and prognosis of lung cancer precise radiotherapy-induced RP.

## Methods

### Patients

A review of pathologically proven lung cancer patients presenting in our hospital between 2008 and May 2013 was performed. 84 patients who had been diagnosed with RP after precise radiotherapy were selected. Enrolled patient characteristics were collected including gender, age, location/pathological type/staging of the tumor, complications, as well as previous treatment history with surgery, radiotherapy, and chemotherapy. Patients who consumed other acid-inhibitory drug or were aged less than 18 were excluded.

### Treatment with Radiotherapy and Chemotherapy

Patient was either treated with radiotherapy alone or combined with chemotherapy. For radiotherapy, intensity-modulated conformal radiotherapy (IMRT) or three-dimensional conformal radiotherapy (3DCRT) is applied. Linear accelerator 6 MVX line was used for routine isocenter irradiation. Radiation regimen was reformulated after radiated to 50 Gy if the lesion was extended [7–9].

For concomitant chemotherapy, *cis*-platinum was used 40 mg/m^2^ week and Taxol was used 60 mg/m^2^ week. Sequenced chemotherapy regime is as follows:

- Taxol 135 mg/m^2^ + *cis*-platinum 80–90 mg/m^2^, navelbine 25 mg/m^2^ d1, d8 + *cis*-platinum 80–90 mg/m^2^, gemcitabine 1,000 mg/m^2^ d1, d8 + *cis*-platinum 80–90 mg/m^2^, or etoposide 50 mg/m^2^ d1–5 + *cis*-platinum 80–90 mg/m^2^, repeated for every 21–28 days.

### Diagnosis and Treatment for Radiation Pneumonitis

Clinical presentations of RP include fatigue, shortness of breath, dry cough/white phlegm, and cough jelly phlegm complicated with low-grade fever if severe. In all cases there was chest distress, chest pain, and dyspnea complicated with protracted course of bacterial infection. Fuzzy shadow or funicular patchy shadows were shown in chest X-ray or CT in accordance with the radiation field [10–12].

Moderate and severe RP with severe symptoms were given high-dose glucocorticoid combined with antibiotics. Usually the dose of glucocorticoid was methylprednisolone 80–120 mg for 1–3 weeks. Then the dose was gradually reduced by 10–50 mg/week. The lowest maintenance dose of glucocorticoid was Medrol (methylprednisolone) at 4–8 mg/day [13–16].

### Patient Grouping

Patients were divided into two groups: PPI group and control group depending on whether they received PPI treatment or not. For PPI group, patients received PPI for 7 days or more after being diagnosed with RP. Three types of PPI were applied: omeprazole, esomeprazole, and lansoprazole. Patients in control group did not receive PPI or any other acid-inhibitory drug. Development and prognosis of RP in two groups were compared, and the effects of different dosages of PPI on development of RP were further investigated. Drug dose was presented as total daily dose/defined daily dose (DDD).

### Evaluation of PR Progression

Blood sample was taken for blood routine and blood gas analysis at various time points during the disease course. Lung injury was followed up by chest X-ray examination. Comparison of related parameters was made between PPI group after at least 7 days of PPI treatment and the control group at the similar time point during the PR course.

### Evaluation of PR Prognosis

The curative effects on RP were evaluated according to ‘Clinical Disease and Curative Standard’ and presented as completed relief (CR), partial relief (PR), stable (SD), and progress (PD). CR + PR was used to generate remission rate.

### Statistical Analysis

Statistical analysis was performed using SPSS 19.0 software. All data are expressed as the mean ± standard deviation (SD). The non-normal distribution of measurement data was measured using nonparametric rank sum test. Enumeration data were presented as rate, and the comparison of rate was used by χ^2^ test. Results were considered as statistically significant when *p* < 0.05.

## Results

### Patient Characteristics

The basic patient characteristics are shown in Table 1. There was no statistically significant difference between the two groups.

**Table 1:** Comparison of basic patient characteristics between two groups [*n* (%)]

Basic characteristics	PPI group (*n* = 57)	Control group (*n* = 27)	*χ* ^2^	*p*
Gender				
Male	35 (61.40)	18 (66.67)	0.218	0.648
Female	22 (38.60)	9 (33.33)		
Mean age (years)				
≥60	19 (33.33)	11 (40.74)	0.438	0.508
<60	38 (66.67)	16 (59.26)		
Pathological type				
Non-small cell carcinoma	54 (94.74)	26 (96.30)	0.098	0.754
Small cell carcinoma	3 (5.26)	1 (3.70)		
Clinical stage				
II	11 (19.30)	7 (25.93)	0.670	0.715
III	28 (49.12)	11 (40.74)		
IV	18 (31.58)	9 (33.33)		
Complicated with cardiovascular disease				
Yes	2 (3.51)	0 (0.00)	0.970	0.325
No	55 (96.49)	27 (0.00)		
Complicated with chronic bronchitis				
Yes	9 (15.79)	4 (14.81)	0.013	0.908
No	48 (84.21)	23 (85.19)		
Diabetes mellitus				
Yes	2 (3.51)	1 (3.70)	0.002	0.964
No	55 (96.49)	26 (96.30)		
Tumor location				
Upper lobe	27 (47.37)	10 (37.04)	2.007	0.367
Middle and lower lobe	13 (22.81)	8 (29.63)		
Hilus of the lung	17 (29.82)	9 (33.33)		
Radiotherapy method				
IMRT	42 (73.68)	19 (70.37)	0.101	0.750
3DCRT	15 (26.32)	8 (29.63)		
Combined with chemotherapy				
Yes	4 (7.02)	1 (3.70)	0.359	0.549
No	53 (92.98)	26 (96.30)		
Surgery				
Yes	13 (22.81)	5 (18.52)	0.200	0.655
No	44 (77.19)	22 (81.48)		

Among 84 patients, there were 53 male cases and 31 female cases with median age of 53 years (ranging from 25 to 78). Majority of patients had non-small cell carcinoma (80/84), and the rest (4/84) had small cell carcinoma. Clinical staging was from II (18/84) to IV (27/84), where 18/84 had surgery and 66/84 did not have surgery. 79/84 patients received radiotherapy alone, while 5/84 received radiotherapy combined with either concomitant chemotherapy or sequenced chemotherapy.

There were 57 patients enrolled in PPI group, and the rest were in control group. For PPI group, PPI drugs were applied as follows: 46 accepted omeprazole, 18 accepted esomeprazole, and 5 accepted lansoprazole. 39 patients only accepted 1 PPI, and 18 patients accepted 2 or more PPI.

### Effects of PPI on PR Progression

Comparison of WBC, blood gas analysis, and chest X-ray results between two groups is shown in Table 2. Compared with control group, PaO_2_ and PCO_2_ had no statistical difference in the PPI group; however, WBC, oxygenation index, blood pH, and lung lesion by chest X-ray were significantly different (*p* < 0.05), indicating that PR in PPI group tended to be severer.

**Table 2:** Comparison of WBC, blood gas analysis, and chest X-ray results between two groups

Group	*n*	WBC > 10 × 10^9^/l or <4 × 10^9^/l [*n* (%)]	PaO_2_ < 60 mmHg [*n* (%)]	Oxygenation index <300 mmHg [*n* (%)]	PCO_2_ > 50 mmHg [*n* (%)]	Blood gas analysis pH < 7.35 pH > 7.45 [*n*(%)]	Increased lesions in lung [*n* (%)]
PPI group	57	33 (57.89)	14 (24.56)	43 (75.44)	21 (36.84)	29 (50.88)	27 (47.37)
Control group	27	6 (22.22)	2 (7.41)	11 (40.74)	14 (51.85)	5 (18.52)	4 (14.81)
χ^2^		9.374	3.496	9.607	1.698	7.963	8.338
*p* value		0.002	0.062	0.002	0.193	0.005	0.004

### Effects of Different Doses of PPI on PR Progression

Among 57 patients in PPI group, 31 cases accepted PPI dose ≤ 1DDD, and 26 cases accepted PPI dose > 1DDD. Comparison of WBC, blood gas analysis, and chest X-ray results between two subgroups of PPI is shown in Table 3. There was no significant difference between the two subgroups.

**Table 3:** Comparison of WBC, blood gas analysis, and chest X-ray results between different doses of PPI

Dose	*n*	WBC > 10 × 10^9^/l or <4×10^9^/l [*n* (%)]	PaO2 < 60 mmHg [*n* (%)]	Oxygenation index <300 mmHg [*n* (%)]	PCO_2_ > 50 mmHg [*n* (%)]	Blood gas analysis pH < 7.35 or >7.45 [*n* (%)]	Increased effusion lesions in lung [*n* (%)]
≤1DDD	31	19 (61.29)	8 (25.81)	22 (70.97)	8 (25.81)	14 (45.16)	13 (41.94)
>1DDD	26	14 (53.85)	7 (26.92)	21 (80.76)	13 (50.00)	15 (57.69)	14 (53.85)
χ^2^		0.321	0.009	0.733	3.557	0.888	0.805
*p* value		0.571	0.924	0.392	0.059	0.346	0.370

### Effect of PPI on PR Prognosis

Effect of PPI on prognosis of the patients in two groups is shown in Table 4. Remission rates between the two groups were statistically significant (*p* < 0.05), with 59.65 % in PPI group and 81.48 % in control group.

**Table 4:** Effect of PPI on PR prognosis [*n* (%)]

Group	*n*	CR	PR	SD	PD	Remission rate (%)
PPI group	57	5 (8.77)	29 (50.88)	15 (26.32)	8 (14.04)	59.65
Control group	27	3 (11.11)	19 (70.37)	3 (11.11)	2 (7.41)	81.48

## Discussion

PPIs are frequently applied to lung cancer patients in treatment of radiotherapy- and/or chemotherapy-induced gastric discomforts. However, recent studies showed that the use of PPI was associated with a slight increase in community-acquired pneumonia and a 30 % increased risk in hospitalized patients [5, 6]. Radiotherapy-induced RP is rather a common complication in lung cancer patients. It usually needs long-term glucocorticoid treatment and is complicated with diseases of other systems [17–19]. So far, there has been no research about the effect of PPI on the development and prognosis of PR. In our study, we compared PPI group and control group of PR patients and found that respiratory dysfunction was statistically greater in PPI group than in control group, and there were more effusion lesions in PPI group as well. In the analysis of PPI effect on prognosis of PR, remission rate in PPI group was 59.65 %, significantly lower than that of control group (81.48 %), indicating that PPI had adverse effects on the development and prognosis of lung cancer precise radiotherapy-induced PR.

The proposed mechanism may be related to the effect that PPI reduces acid production and, therefore, creates a favorable environment for bacterial overgrowth in the stomach and esophagus and increases the risk of bacterial aspiration. Recent study showing that pantoprazole, one of PPIs, decreases gastroesophageal muscle tone in newborn rats seems to provide further support for this hypothesis [20]. Researchers have also proved in vitro that acid-inhibitory drugs can affect the functions of neutrophil and natural killer cell, weakening pathogenic bacteria elimination [21–24].

In the analysis of different dosages of PPIs, we did not see any significant difference between RP development and prognosis. This was consistent with the results of Gulmez et al. [25], who considered that there was no obvious relation between PPI dose and pneumonia. However, several researchers have reported that with the higher PPI dose, there was greater possibility that pneumonia occurred, and along with the treatment time, the correlation became weaker [26–28]. Compared to hospital-acquired pneumonia, RP is more severe and complicated; patients have high probability of stomach mucosa damage, and medication dose varies, which make it difficult to explore the effect of different PPI dosages on RP.

In summary, medical principles that weigh the advantages and disadvantages of PPI should be complied with in clinical resolution of PPI-preventive application in lung cancer precise radiotherapy-induced RP. PPIs should be utilized cautiously to avoid the adverse effects of PPI in lung cancer patients.

## References

[1] Jemal A (2011). Global cancer statistics. CA: A Cancer Journal for Clinicians.

[2] Vogelius IR, Bentzen SM (2012). A literature-based meta-analysis of clinical risk factors for development of radiation induced pneumonitis. Acta Oncologica.

[3] Palma DA, Senan S, Tsujino K, Barriger RB, Rengan R, Moreno M, Bradley JD, Kim TH, Ramella S, Marks LB (2013). Predicting radiation pneumonitis after chemoradiation therapy for lung cancer: An international individual patient data meta-analysis. International Journal of Radiation Oncology Biology Physics.

[4] Coia LR, Myerson RJ, Tepper JE (1995). Late effects of radiation therapy on the gastrointestinal tract. International Journal of Radiation Oncology Biology Physics.

[5] Meijvis SC, Cornips MC, Voorn GP, Souverein PC, Endeman H, Biesma DH, Leufkens HG, van de Garde EM (2011). Microbial evaluation of proton-pump inhibitors and the risk of pneumonia. The European Respiratory Journal.

[6] Eom CS, Jeon CY, Lim JW, Cho EG, Park SM, Lee KS (2011). Use of acid-suppressive drugs and risk of pneumonia: A systematic review and meta-analysis. CMAJ.

[7] Marks LB, Spencer DP, Sherouse GW, Bentel G, Clough R, Vann K, Jaszczak R, Coleman RE, Prosnitz LR (1995). The role of three dimensional functional lung imaging in radiation treatment planning: The functional dose-volume histogram. International Journal of Radiation Oncology Biology Physics.

[8] De Ruysscher D, Faivre-Finn C, Nestle U, Hurkmans CW, Le Pechoux C, Price A, Senan S (2010). European Organisation for Research and Treatment of Cancer recommendations for planning and delivery of high-dose, high-precision radiotherapy for lung cancer. Journal of Clinical Oncology.

[9] De Ruysscher D, Kirsch CM (2010). PET scans in radiotherapy planning of lung cancer. Radiotherapy and Oncology.

[10] Mac Manus MP, Ding Z, Hogg A, Herschtal A, Binns D, Ball DL, Hicks RJ (2011). Association between pulmonary uptake of fluorodeoxyglucose detected by positron emission tomography scanning after radiation therapy for non-small-cell lung cancer and radiation pneumonitis. International Journal of Radiation Oncology Biology Physics.

[11] Guckenberger M, Baier K, Polat B, Richter A, Krieger T, Wilbert J, Mueller G, Flentje M (2010). Dose-response relationship for radiation-induced pneumonitis after pulmonary stereotactic body radiotherapy. Radiotherapy and Oncology.

[12] Roeder F, Friedrich J, Timke C, Kappes J, Huber P, Krempien R, Debus J, Bischof M (2010). Correlation of patient-related factors and dose-volume histogram parameters with the onset of radiation pneumonitis in patients with small cell lung cancer. Strahlentherapie und Onkologie.

[13] Barriger RB, Fakiris AJ, Hanna N, Yu M, Mantravadi P, McGarry RC (2010). Dose-volume analysis of radiation pneumonitis in non-small-cell lung cancer patients treated with concurrent cisplatinum and etoposide with or without consolidation docetaxel. International Journal of Radiation Oncology Biology Physics.

[14] Borst GR, Ishikawa M, Nijkamp J, Hauptmann M, Shirato H, Onimaru R, van den Heuvel MM, Belderbos J, Lebesque JV, Sonke JJ (2009). Radiation pneumonitis in patients treated for malignant pulmonary lesions with hypofractionated radiation therapy. Radiotherapy and Oncology.

[15] Shi A, Zhu G, Wu H, Yu R, Li F, Xu B (2010). Analysis of clinical and dosimetric factors associated with severe acute radiation pneumonitis in patients with locally advanced non-small cell lung cancer treated with concurrent chemotherapy and intensity-modulated radiotherapy. Radiation Oncology.

[16] Dang J, Li G, Lu X, Yao L, Zhang S, Yu Z (2010). Analysis of related factors associated with radiation pneumonitis in patients with locally advanced non-small-cell lung cancer treated with three-dimensional conformal radiotherapy. Journal of Cancer Research and Clinical Oncology.

[17] Mak RH, Alexander BM, Asomaning K, Heist RS, Liu CY, Su L, Zhai R, Ancukiewicz M, Napolitano B, Niemierko A (2012). A single-nucleotide polymorphism in the methylene tetrahydrofolate reductase (MTHFR) gene is associated with risk of radiation pneumonitis in lung cancer patients treated with thoracic radiation therapy. Cancer.

[18] Tucker SL, Jin H, Wei X, Wang S, Martel MK, Komaki R, Liu HH, Mohan R, Chen Y, Cox JD (2010). Impact of toxicity grade and scoring system on the relationship between mean lung dose and risk of radiation pneumonitis in a large cohort of patients with non-small cell lung cancer. International Journal of Radiation Oncology Biology Physics.

[19] Yamashita H, Nakagawa K, Nakamura N, Koyanagi H, Tago M, Igaki H, Shiraishi K, Sasano N, Ohtomo K (2007). Exceptionally high incidence of symptomatic grade 2–5 radiation pneumonitis after stereotactic radiation therapy for lung tumors. Radiation Oncology.

[20] Welsh, C., Kasirer, M. Y., Pan, J., Shifrin, Y., & Belik, J. (2014). Pantoprazole decreases gastroesophageal muscle tone in newborn rats via rho-kinase inhibition. *Am J Physiol Gastrointest Liver Physiol*. doi:10.1152/ajpgi.00005.2014. 10.1152/ajpgi.00005.2014 24699328

[21] Sibbing D, Morath T, Stegherr J, Braun S, Vogt W, Hadamitzky M, Schomig A (2009). antiplatelet effects of clopidogrel. Thrombosis and Haemostasis.

[22] Ho PM, Maddox TM, Wang L, Fihn SD, Jesse RL, Peterson ED, Rumsfeld JS (2009). Risk of adverse outcomes associated with concomitant use of clopidogrel and proton pump inhibitors following acute coronary syndrome. JAMA: The Journal of the American Medical Association.

[23] Corley DA, Kubo A, Zhao W, Quesenberry C (2010). Proton pump inhibitors and histamine-2 receptor antagonists are associated with hip fractures among at-risk patients. Gastroenterology.

[24] Charlot M, Ahlehoff O, Norgaard ML, Jorgensen CH, Sorensen R, Abildstrom SZ, Hansen PR, Madsen JK, Kober L, Torp-Pedersen C (2010). Proton-pump inhibitors are associated with increased cardiovascular risk independent of clopidogrel use: A nationwide cohort study. Annals of Internal Medicine.

[25] Gulmez SE, Holm A, Frederiksen H, Jensen TG, Pedersen C, Hallas J (2007). Use of proton pump inhibitors and the risk of community-acquired pneumonia: A population-based case-control study. Archives of Internal Medicine.

[26] Laheij RJ, Sturkenboom MC, Hassing RJ, Dieleman J, Stricker BH, Jansen JB (2004). Risk of community-acquired pneumonia and use of gastric acid-suppressive drugs. JAMA: The Journal of the American Medical Association.

[27] Dublin S, Walker RL, Jackson ML, Nelson JC, Weiss NS, Jackson LA (2010). Use of proton pump inhibitors and H2 blockers and risk of pneumonia in older adults: A population-based case–control study. Pharmacoepidemiology and Drug Safety.

[28] Luna CM, Blanzaco D, Niederman MS, Matarucco W, Baredes NC, Desmery P, Palizas F, Menga G, Rios F, Apezteguia C (2003). Resolution of ventilator-associated pneumonia: Prospective evaluation of the clinical pulmonary infection score as an early clinical predictor of outcome. Critical Care Medicine.

